# A Multi-Modal Light Sheet Microscope for High-Resolution 3D Tomographic Imaging with Enhanced Raman Scattering and Computational Denoising

**DOI:** 10.3390/s25082386

**Published:** 2025-04-09

**Authors:** Pooja Kumari, Björn Van Marwick, Johann Kern, Matthias Rädle

**Affiliations:** 1 CeMOS Research and Transfer Center, Mannheim University of Applied Sciences, 68163 Mannheim, Germany; b.vanmarwick@hs-mannheim.de (B.V.M.); m.raedle@hs-mannheim.de (M.R.); 2 Medical Faculty Mannheim, Heidelberg University, 68167 Mannheim, Germany; johann.kern@medma.uni-heidelberg.de

**Keywords:** raman tomography, multi-modal imaging, Deep Image Prior (DIP), light sheet microscopy, fluorescence suppression, 3D molecular imaging, super-resolution

## Abstract

Three-dimensional (3D) cellular models, such as spheroids, serve as pivotal systems for understanding complex biological phenomena in histology, oncology, and tissue engineering. In response to the growing need for advanced imaging capabilities, we present a novel multi-modal Raman light sheet microscope designed to capture elastic (Rayleigh) and inelastic (Raman) scattering, along with fluorescence signals, in a single platform. By leveraging a shorter excitation wavelength (532 nm) to boost Raman scattering efficiency and incorporating robust fluorescence suppression, the system achieves label-free, high-resolution tomographic imaging without the drawbacks commonly associated with near-infrared modalities. An accompanying Deep Image Prior (DIP) seamlessly integrates with the microscope to provide unsupervised denoising and resolution enhancement, preserving critical molecular details and minimizing extraneous artifacts. Altogether, this synergy of optical and computational strategies underscores the potential for in-depth, 3D imaging of biomolecular and structural features in complex specimens and sets the stage for future advancements in biomedical research, diagnostics, and therapeutics.

## 1. Introduction

Three-dimensional (3D) cell cultures, especially spheroids, more faithfully recapitulate the structural, biochemical, and functional attributes of native tissues than traditional two-dimensional (2D) monolayer cultures [1,2]. Such multilayered constructs are highly valuable for interrogating tumor microenvironment dynamics, drug responses, and intercellular communication—central topics in oncology and tissue engineering. Yet, their inherent complexity and thickness demand high-resolution, label-free imaging techniques that preserve the intrinsic state of the samples, a requirement not readily fulfilled by many conventional methods [3,4,5,6,7,8,9,10,11,12].

**Figure 1:**
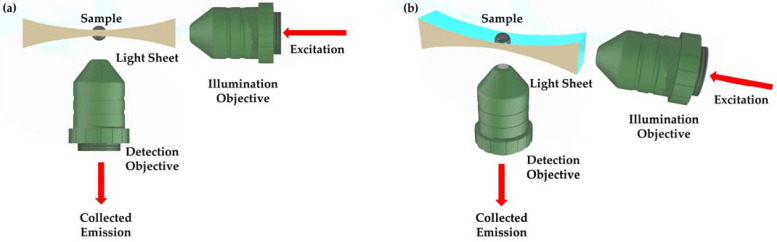
Schematic representation of the principle of light sheet microscopy: (**a**) Top View, (**b**) Side View. The illumination and detection axes are oriented orthogonally, with the sample positioned at their intersection. A laser beam is shaped into a thin light sheet to provide selective plane illumination within the focal plane of the detection objective. The detection objective captures the illuminated plane and projects it onto the camera sensor, facilitating high-resolution imaging of the targeted section. This approach ensures efficient illumination and precise detection [18].

Light sheet microscopy (LSM) meets these needs by using a thin sheet of laser light to illuminate only the focal plane (Figure 1), thereby minimizing out-of-focus excitation and reducing phototoxic effects [13,14,15]. In contrast to point-scanning approaches, LSM acquires an entire image plane in a single exposure, which significantly expedites volumetric data collection and lowers the risk of photo bleaching [16]. Although fluorescence has been the primary contrast mechanism in many LSM implementations, capturing Rayleigh and Raman scattering signals within the same instrument can provide both morphological and molecular insights [17]. Rayleigh scattering arises from refractive index discontinuities in cellular structures, while Raman scattering records the vibrational fingerprints of biomolecules, obviating the need for exogenous labels.

Despite advancements in light sheet Raman microscopy (LSRM), many systems continue to rely on near-infrared (NIR) excitation wavelengths (e.g., 785 nm) to minimize autofluorescence [18]. While effective in reducing background interference, this approach diminishes Raman scattering cross-sections and electronic sensitivities and compromises spatial resolution, limiting the quality and speed of 3D reconstructions. Addressing these limitations, a novel multi-modal 3D imaging setup was developed, utilizing visible excitation to enhance Raman scattering efficiency while suppressing background fluorescence. To optimize performance, our platform integrates acousto-optic tunable filters (AOTFs) for precise and agile spectral selection paired with advanced deep learning algorithms for image enhancement. Among these, the Deep Image Prior (DIP) algorithm has been specifically chosen for its superior performance in light sheet microscopy, consistently surpassing other self-supervised and unsupervised methods such as ZS-DeconvNet, Noise2Noise, and Noise2Void. DIP effectively combines noise suppression, correction of diffraction-induced artifacts, and preservation of structural integrity, resulting in artifact-free volumetric reconstructions with exceptional molecular and spatial fidelity. This integration of visible excitation with DIP establishes a transformative framework for achieving high-resolution, high-speed 3D imaging, setting a new standard in the field of light sheet Raman microscopy (LSRM) [18,19,20,21,22,23,24,25,26].

By integrating Rayleigh, Raman, and fluorescence signals into a single experimental workflow, the newly developed system generates comprehensive hyperspectral datasets without the need for additional labels, ensuring that the physiological state of spheroids remains unaltered. Critically, the shift to visible wavelengths delivers stronger Raman signals and higher imaging throughput than comparable NIR-driven modalities. Taken together, this novel light sheet microscope platform builds on a previously described microscope setup that included a 785 nm IR-range laser [20]; however, Raman imaging using this laser did not yield meaningful information. Consequently, the new system overcomes the limitations of the previous design, enabling more detailed and accurate studies in tumor biology, tissue modeling, and drug discovery, while advancing the boundaries of noninvasive, high-resolution 3D molecular imaging for complex biological systems.

## 2. Materials and Methods

This study presents a multi-modal light sheet microscope (LSM) designed to address the challenges of traditional Raman imaging, including weak scattering efficiency, fluorescence interference, and limited spatial resolution. The LSM integrates Raman scattering, Rayleigh scattering, and fluorescence imaging modalities supported by a dual-wavelength excitation system to enhance Raman efficiency while ensuring deep tissue penetration and minimal photodamage. The detailed design, experimental setup, and protocols are provided below to ensure replicability and facilitate future advancements.

### 2.1. Measurement Setup Design

**Figure 2:**
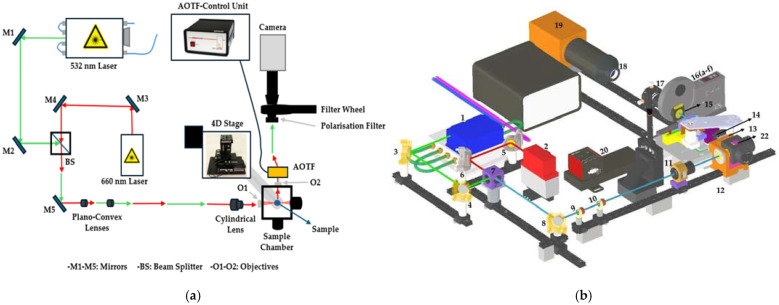
The Raman light sheet microscope with 532 nm and 660 nm lasers and sCMOS camera. (**a**) Top-down schematic and (**b**) CAD Model of the Raman Light Sheet Microscope in Isometric View. Illumination path includes lasers, broadband mirrors, dichroic mirror, spherical (f = 25 mm, 50 mm) and cylindrical (f = 50 mm) lenses, and a 10× illumination objective. Detection path features a 20× detection objective, AOTF, polarization and filter wheels, tube lens, and sCMOS camera. The system includes a precision positioning stage and sample chamber. See Table 1 for component details.

Figure 2 shows the schematic and the 3D illustration of the experimental setup used for the multi-modal light sheet microscopy. Figure 2a depicts the optical path for both 532 nm and 660 nm lasers. The laser beams are directed through mirrors (M1-M5) and a beam splitter (BS) to focus the light onto the sample using a cylindrical lens. The system is equipped with a 4D stage for precise sample movement and a camera to capture the images. The Acousto-Optic Tunable Filter (AOTF) is used for wavelength selection, while a polarization filter and filter wheel help manage light interference and background. Figure 2b shows a detailed 3D rendering of the setup, highlighting key components, such as the lasers, optical elements, and the sample chamber. The CAD model was manually adjusted for clearer visualization and may not reflect the exact physical positions of components used in the experimental setup. The diagram helps visualize the complex optical arrangement and how the system enables the simultaneous collection of multiple imaging modalities (Raman, Rayleigh, and fluorescence), supporting comprehensive analysis of biological samples with high precision. This is achieved through rapid, sequential switching between modalities enabled by the AOTF, eliminating the need for physical realignment and preserving spatial calibration across datasets. Also, a detailed explanation of the system components is stated in Table 1, and their role in image acquisition can be found in the subsequent Section 2.1.

#### 2.1.1. Excitation Lasers

The LSM utilizes two continuous-wave (CW) lasers operating at wavelengths of 532 nm and 660 nm (λ_1_ = 532 nm, λ_2_ = 660 nm). The 532 nm laser, with a power range of 0–2 W, was selected for its high Raman scattering efficiency. This efficiency is governed by the relationship:

(1) $$\sigma_{R}\propto \left(\frac{1}{\lambda^{4}}\right)I\left(\upsilon \right){\left|\alpha \right|}^{2}$$

where $\sigma_{R}$ is the Raman scattering cross-section, λ is the excitation wavelength, $I\left(\upsilon \right)$ is the vibrational intensity at frequency υ, and α is the polarizability tensor. The 660 nm laser, operating at 0–130 mW, was chosen to penetrate deeper tissue layers while mitigating autofluorescence. This dual-wavelength strategy enables comprehensive imaging from surface structures to deep tissue regions with high sensitivity.

#### 2.1.2. Beam Shaping and Light Sheet Formation

The illumination system projects a vertically oriented light sheet into the sample chamber, thereby ensuring uniform illumination while minimizing scattering artifacts. The axial resolution—defined by the beam waist—was experimentally determined to be approximately 8 µm for both 532 nm and 660 nm excitation lasers using a BP209-VIS/M Scanning-Slit Optical Beam Profiler (Thorlabs Inc., Newton, MA, USA). This configuration enables high-precision optical sectioning at 10 µm increments along the optical axis, an essential capability for capturing subcellular structures in sequential imaging. Furthermore, the 635 µm × 635 µm effective field of view accommodates spheroidal samples of comparable diameter, facilitating comprehensive volumetric sectioning across diverse experimental contexts. These parameters collectively ensure high-fidelity, multi-modal Raman imaging, especially when combined with reliable sample positioning that preserves accurate alignment throughout sequential acquisitions. For beam conditioning, the laser output was first collimated and expanded by a Keplerian telescope providing a magnification factor:

(2) $$M=\frac{f1}{f2}$$

where f1 and f2 are the focal lengths of the input and output lenses, respectively. The expanded beam was shaped into a static light sheet using a cylindrical lens yielding a Gaussian intensity profile described by:

(3) $$I\left(x,z\right)=I_{0}exp\left(\frac{-{2x}^{2}}{w_{0}^{2}}\right).{sinc}^{2}\left(\frac{z}{z_{R}}\right)$$

where $I_{0}$ is the peak intensity, $w_{0}$ is the beam waist radius, x is the transverse axis, z is the axial axis, and $z_{R}$ is the Rayleigh range. The axial propagation of the beam was characterized by:

(4) $$w\left(z\right)=w_{0}\sqrt{1+{\left(\frac{z}{z_{R}}\right)}^{2}}$$

where $w\left(z\right)$ represents the beam width at a given axial distance z.

#### 2.1.3. Spectral Filtering and Detection System

Modular Detection and Filtering System: The detection framework employs a high-performance scientific Complementary Metal-Oxide-Semiconductor (sCMOS) camera integrated with a versatile optical filter assembly optimized for multimodal imaging applications. This assembly comprises an Acousto-Optic Tunable Filter (AOTF), polarization filters, and a combination of longpass, notch, and shortpass filters (Figure 2). For clearer component details, the numbered items in Figure 2b are referenced in Table 1. The AOTF facilitates precise spectral selection, enabling the fine-tuning of transmitted wavelengths for Rayleigh, Raman, and fluorescence modalities without necessitating physical realignment of the optical components. The operational principle of the AOTF is described by the equation:

(5) $$\lambda_{c}=\frac{\nu }{nf}$$

where $\lambda_{c}$ denotes the central wavelength, ν is the acoustic velocity within the AOTF material, n represents the refractive index, and f is the acoustic frequency applied. This relationship allows for dynamic and accurate control of the spectral filtering properties essential for multimodal imaging applications.

Polarization filters are employed to enhance image contrast by selectively rejecting undesired polarization states of incoming light, particularly those resulting from multiple scattering events, which introduce depolarized light components that can degrade spectral fidelity and obscure weak Raman signals. By eliminating these unwanted states, the system significantly improves the signal-to-noise ratio, enhancing the efficiency and sensitivity of inelastic scattering detection. While some light loss occurs due to polarization filtering, it remains minimal and does not significantly impact imaging performance, as the system is optimized for high-sensitivity detection, ensuring that the benefits of improved contrast and spectral precision outweigh any minor intensity reduction. Additionally, the integration of longpass, notch, and shortpass filters further refines spectral filtering, allowing for the isolation of specific wavelength ranges pertinent to the different imaging modalities, ensuring optimal contrast and signal clarity.

For Raman imaging at 660 nm and 532 nm, Filter 1 (660 nm longpass) and Filter 6 (532 nm longpass) were used, respectively, as listed in Table 1. These settings were selected due to their superior performance in suppressing Rayleigh scattering and preserving high-quality Stokes-shifted Raman signals. No additional filters were applied for Rayleigh imaging, allowing for direct acquisition of elastically scattered light. For a summary of the effective spectral configurations across modalities, please refer to Table 2.

This modular detection and filtering system enables rapid and precise adjustments to the spectral parameters, ensuring optimal performance across various imaging conditions. The combination of AOTF-based spectral tuning and polarization filtering minimizes background noise and optical distortions, thereby facilitating high-fidelity Raman spectroscopy and fluorescence imaging. Consequently, this configuration supports robust and reproducible multi-modal imaging essential for detailed biochemical and structural analyses in complex biological samples.

High-Resolution Detection: Scattered and emitted photons were orthogonally collected using a high numerical aperture (NA = 1.2) objective lens, thereby achieving lateral and axial resolutions as delineated by the Rayleigh criterion:

(6) $$\Delta r=0.61\frac{\lambda }{NA}$$

where Δr represents the spatial resolution and λ is the detection wavelength (AOTF) of the emitted or scattered photons. The configuration achieved lateral and axial resolutions of ~250 nm and ~600 nm, respectively. The detection system employed a scientific CMOS (sCMOS) camera (Hamamatsu ORCA Flash 4.0) with a 16-bit dynamic range to capture the scattered light. The pixel pitch of 6.5 µm ensured Nyquist sampling based on the optical resolution of the system. This configuration enables precise spatial and spectral discrimination essential for detailed biochemical and structural analyses within complex biological samples.

#### 2.1.4. Calibration and System Validation

Polystyrene microspheres (1 µm diameter) were used to validate light sheet thickness and uniformity through Gaussian intensity fitting. The effective full-width at half-maximum (FWHM) thickness was optimized to ~2 µm. The uniformity and thickness of the light sheet were assessed by imaging fluorescent beads distributed in a transparent medium. Silicon wafers ($\nu =532\;{cm}^{-1}$) served as spectral references, with a Raman peak accuracy maintained within $\pm 2\;{cm}^{-1}$.

### 2.2. Sample Preparation and Alignment

#### 2.2.1. Sample Preparation

Biological specimens, encompassing three-dimensional (3D) spheroids and adherent cell cultures, were meticulously prepared using standardized protocols to ensure optimal imaging fidelity and preserve cellular integrity. Spheroids were encapsulated within a low-scattering hydrogel matrix, which afforded high optical clarity and maintained physiological conditions throughout the imaging process. This embedding strategy effectively reduced light scattering, thereby enhancing image quality and sustaining a supportive environment for cellular function.

For the purposes of this investigation, 3D spheroids were generated from two HPV-negative head and neck squamous cell carcinoma (HNSCC) cell lines: UMSCC-14C originating from oral cavity carcinoma. Both cell lines were cultured in Eagle’s Minimum Essential Medium (EMEM) supplemented with 10% fetal bovine serum (FBS) and 1% penicillin/streptomycin. Cultures were maintained under standard conditions at 37 °C in a humidified atmosphere containing 5% CO_2_. Cells were detached enzymatically using trypsin/EDTA, quantified with a Neubauer hemocytometer, and subsequently seeded into ultra-low attachment (ULA) 96-well plates at densities of 2.5 × 10^4^ or 5 × 10^4^ cells per well to promote spheroid formation. These spheroids were cultured for up to eight days, with medium replenishments on days 3, 5, and 8, ultimately achieving diameters ranging from 300 to 400 µm.

To evaluate the impact of chemotherapeutic intervention, spheroids were treated with cisplatin at a concentration of 100 µm on the fourth day of culture, whereas control spheroids received an equivalent volume of dimethyl sulfoxide (DMSO). Following a 72-h incubation period, both treated and untreated spheroids were fixed using 4% formalin to preserve structural integrity for subsequent imaging analyses. The fixed samples were then mounted into a bespoke, 3D-printed hydrogel carrier designed to facilitate precise alignment with the light-sheet illumination and detection optics. This setup enabled multi-view imaging under physiological conditions (37 °C and 5% CO_2_), ensuring the viability and structural preservation of the samples throughout extended imaging sessions.

Another spheroid, HT29 (colorectal adenocarcinoma) cells, were cultured in Dulbecco’s Modified Eagle Medium (DMEM) supplemented with 10% fetal bovine serum (FBS) and 1% Penicillin/Streptomycin. Cells were enzymatically detached, quantified, and seeded under identical conditions in ULA 96-well plates at densities of 5 × 10^4^ cells per well to generate spheroids. HT29 spheroids were cultured in the same incubation conditions as UM-SCC-14C and exhibited a compact morphology within 72 h, reaching diameters of 300–400 µm over an eight-day period.

Both UM-SCC-14C and HT29 spheroids were prepared under identical conditions for Raman light sheet microscopy (RLSM) imaging, ensuring uniform sample integrity across experiments.

#### 2.2.2. Hydrogel Embedding

Fixed tumor spheroids were embedded within a low-melting-point agarose matrix to ensure positional stability during imaging procedures. This agarose gel effectively mimics the extracellular environment, providing essential structural support and maintaining the integrity of the samples throughout the imaging process. By replicating physiological conditions, the agarose embedding preserves the native architecture of the spheroids, facilitating high-fidelity imaging and accurate morphological assessments.

#### 2.2.3. Positioning System

Samples were mounted within a custom-fabricated, three-dimensional (3D) printed holder engineered for enhanced stability and multi-view imaging capabilities. This holder was affixed to a precision 4D stage, which provides translational resolution of 1.5 µm along the X, Y, and Z axes and rotational adjustments with a precision of 0.5 degrees. This sophisticated configuration enabled meticulous alignment of the sample with the light sheet, effectively minimizing motion artifacts and ensuring high reproducibility in imaging.

### 2.3. Imaging Workflow and Computational Processing

Multi-modal imaging was conducted using 532 nm and 660 nm lasers, with Raman imaging optimized at laser powers of 130 mW and 350 mW and a prolonged exposure time of 5000 ms to maximize signal quality. Rayleigh scattering images were captured at 100 ms exposure using 1 mW laser powers to ensure optimal contrast. Z-stacks were acquired at 10 µm intervals along the vertical axis for high-resolution three-dimensional reconstruction. The Acousto-Optic Tunable Filter (AOTF) provided precise spectral alignment across modalities, eliminating manual realignment (Table 2). This setup allowed for rapid sequential acquisition of Raman, Rayleigh, and fluorescence images within a single imaging session, minimizing temporal and spatial misalignment between modalities. However, as the primary focus of this study is on Raman imaging, Rayleigh and fluorescence data are used solely for complementary structural validation. Although the optical system provides a consistent field of view across modalities (~635 µm × 635 µm), images presented in the Results section may appear with variable dimensions due to post-acquisition cropping for enhanced contrast and clearer visualization of regions of interest. These adjustments do not reflect differences in imaging scale.

Raman spectra were recorded using a Tec5 spectrometer for 660 nm and a Kaiser spectrometer for 532 nm lasers. Hyperspectral Raman data were stored as spectral cubes for biochemical profiling, while structural images were saved in .tiff format for downstream processing.

Raman spectra were analyzed for key vibrational modes, and the corresponding molecular assignments are detailed in Table 3. These features were integral for determining the biochemical changes and structural characteristics of the sample. Peak intensity ratios were analyzed to evaluate molecular alterations, such as those induced by cisplatin treatment.

Rayleigh scattering images, which lack spectral data, were denoised using the Deep Image Prior (DIP) algorithm, an unsupervised framework leveraging convolutional neural networks (CNNs) to suppress noise while preserving spatial features. DIP aims to recover the underlying clean image X by optimizing the CNN $f_{\theta }\left(z\right)$, where z is a fixed random input. The optimization objective is expressed as:

(7) $$\hat{\theta }=arg\underset{\theta }{min}{\left\|f_{\theta }\left(z\right)-Y\right\|}^{2}$$

**Figure 3:**
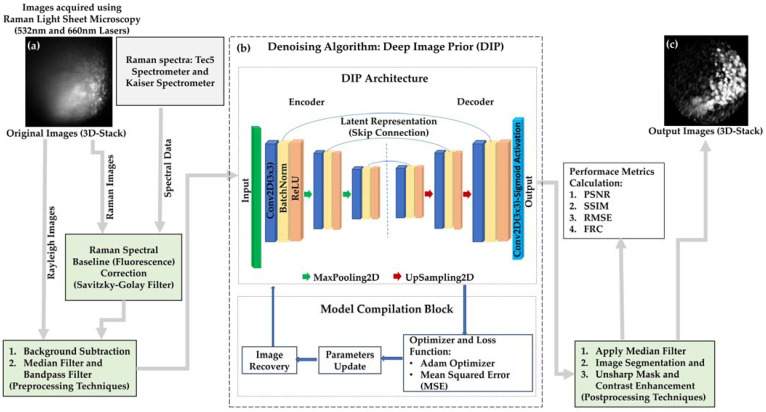
Denoising Workflow Using the DIP Algorithm. (**a**) Original images acquired with multi-modal Raman light sheet microscopy. (**b**) Implementation of the DIP algorithm, including its architecture and model compilation methods, following preprocessing and postprocessing techniques. (**c**) Denoised outputs were evaluated using quantitative metrics (PSNR, SSIM, RMSE, and FRC) (Note: The displayed image regions are cropped from the original 635 µm × 635 µm field of view for clarity).

Here, $f_{\theta }\left(z\right)$ is the CNN with learnable weights θ, Y represents the observed noisy image, which serves as the target during optimization, and z is typically a fixed random noise input. DIP uses a fixed random noise input, with no pretraining on external datasets. Instead, it learns to denoise directly during optimization. The encoder-decoder CNN architecture incorporates skip connections to preserve fine details, while the decoder reconstructs the image from a compressed representation. A sigmoid activation constrains the output pixel values between 0 and 1 (Figure 3).

To complement the DIP framework, a systematic pre- and post-processing pipeline was applied to improve image quality while preserving structural fidelity. Background subtraction was performed using a reference image to normalize intensity profiles. Additive and subtractive Gaussian noise was introduced to generate corrupted pairs, facilitating the network’s learning of robust, noise-resistant features. Median filtering helped suppress high-frequency noise without blurring structural details. A circular region of interest (ROI), defined using skimage’s disk function, localized analysis to biologically relevant areas while minimizing edge artifacts. Adaptive histogram equalization improved local contrast within the ROI, and morphological operations such as dilation and erosion were employed to refine small structures. These steps were optimized to align with DIP’s self-regularizing nature, enhancing its ability to produce clean and structurally accurate reconstructions. DIP denoising was applied on a per-slice basis for volumetric Raman imaging, ensuring localized enhancement while preserving axial continuity. Its self-regularizing nature inherently suppresses acquisition-induced noise while maintaining structural fidelity. To prevent overfitting to noise, early stopping criteria were implemented, and performance was validated using PSNR, SSIM, and RMSE metrics. Unlike traditional methods, DIP does not require external training data, instead relying on the internal image statistics captured by the CNN architecture. This makes it particularly effective for Raman light sheet microscopy, where artifacts from diffraction and non-uniform illumination can degrade image quality.

Raman spectra were baseline-corrected using the Savitzky–Golay algorithm to eliminate fluorescence artifacts, ensuring accurate peak identification. The DIP algorithm was applied to both Raman images and spectral data, enhancing spatial fidelity and maintaining the integrity of spectral signatures.

The efficacy of the denoising workflow was validated using quantitative metrics as explained above. The effectiveness of the denoising and image enhancement algorithms was evaluated using key quantitative metrics to ensure noise reduction and structural preservation. Peak Signal-to-Noise Ratio (PSNR) values between 30–50 dB indicated effective noise suppression, with values closer to 50 dB reflecting near-perfect image restoration suitable for resolving fine subcellular details [30,31,32,33]. Structural Similarity Index (SSIM) values in the range of 0.90–0.99 confirmed the preservation of luminance, contrast, and structural integrity in the denoised images [34,35,36]. Root Mean Squared Error (RMSE) values below 0.05 demonstrated minimal pixel-wise deviations, ensuring accurate reconstruction [37,38,39]. Fourier Ring Correlation (FRC) values between 0.7 and 1.0 highlighted the retention of high-frequency spatial details essential for sharpness and resolution [40,41].

(8) $$PSNR=10{log}_{10}\left(\frac{{MAX}^{2}}{MSE}\right)$$

where MAX is the maximum possible pixel value of the image and MSE is the mean squared error between the original and processed image.

(9) $$SSIM\left(x,y\right)=\frac{\left(2\mu_{x}\mu_{y}+C_{1}\right)\left({2\sigma }_{xy}+C_{2}\right)}{\left(\mu_{x}^{2}+\mu_{y}^{2}+C_{1}\right)\left(\sigma_{x}^{2}+\sigma_{y}^{2}+C_{2}\right)}$$

where $\mu_{x}$ and $\mu_{y}$ are the mean intensities of images x and y. $\sigma_{x}^{2}$ and $\sigma_{y}^{2}$ are the variances of the images x and y, respectively. $\sigma_{xy}$ is the covariance between the images. $C_{1}$ and $C_{2}$ are constants to stabilize the division when the denominator is close to zero.

(10) $$RMSE=\sqrt{\frac{1}{N}\sum_{i=1}^{N}{\left(I_{original,i}-I_{denoised,i}\right)}^{2}}$$

where N is the total number of pixels in the image. $I_{original}$ and $I_{denoised}$ are the pixel intensities in the original and deblurred images, respectively.

(11) $$FRC=\frac{\sum_{i\epsilon R\left(f\right)}{FFT}_{1}\left(i\right).\bar{{FFT}_{2}\left(i\right)}}{\sqrt{\sum_{i\epsilon R\left(f\right)}{FFT}_{1}\left(i\right)\vee^{2}.\sum_{i\epsilon R\left(f\right)}{FFT}_{1}\left(i\right)\vee^{2}}}$$

where ${FFT}_{1}$ and ${FFT}_{2}$ are the Fourier transforms of the original and denoised images. $R\left(f\right)$ represents the pixels corresponding to the frequency f.

These metrics were computed between the original noisy Raman images and their corresponding DIP-enhanced outputs, referred to as $I_{original}$ and $I_{denoised}$, respectively. The DIP framework operates in a self-supervised manner, using the noisy image as both input and target. Prior to training, images were background-subtracted, converted to grayscale, and denoised with a median filter. The DIP model was trained slice-wise with early stopping to avoid overfitting. This pipeline contributed to the high signal fidelity and the quantitative improvements reflected in the reported metrics.

Collectively, these metrics validated the algorithms’ ability to produce high-quality microscopy images while maintaining the accuracy of biological structures.

## 3. Results

### 3.1. Comparative Analysis of Cisplatin-Induced Structural and Molecular Alterations in Spheroids Using 660 nm and 532 nm Excitation

**Figure 4:**
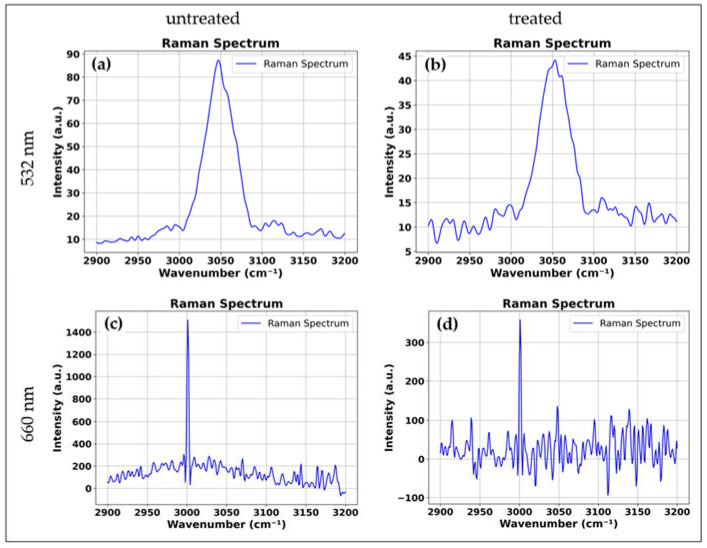
(**a**) Raman spectra at 532 nm untreated, (**b**) treated, and (**c**) at 660 nm untreated, (**d**) treated.

Light sheet Raman microscopy (LSRM) was employed to investigate structural and biochemical alterations in cisplatin-treated spheroids, leveraging 532 nm and 660 nm excitation for a comparative analysis of Raman scattering efficiency, penetration depth, and autofluorescence interference. The excitation wavelength in Raman light sheet microscopy directly affects these parameters, influencing molecular specificity and imaging depth. 532 nm excitation enhances Raman signal intensity due to its higher scattering cross-section but introduces strong autofluorescence, reducing spectral contrast and complicating molecular interpretation. Conversely, 660 nm excitation minimizes fluorescence interference, improving biochemical selectivity, but exhibits lower Raman cross-section, leading to decreased signal intensity and requiring longer acquisition times. These findings emphasize the trade-off between signal strength and fluorescence suppression, necessitating wavelength optimization for high-fidelity volumetric Raman imaging in drug-response studies. Figure 4 presents Raman spectral analysis of untreated and cisplatin-treated spheroids under 532 nm and 660 nm excitation, focusing on the 2990–3200 cm^−1^ region corresponding to C-H stretching vibrations in lipids and proteins.

At 532 nm excitation (Figure 4a,b), untreated spheroids exhibit a strong Raman signal intensity due to the higher scattering efficiency of the shorter wavelength. However, autofluorescence interference is evident, requiring spectral correction. Cisplatin-treated spheroids (Figure 4b) show a noticeable decrease in peak intensity at 3030 cm^−1^, indicating lipid degradation and protein denaturation, consistent with apoptosis-induced structural changes.

At 660 nm excitation (Figure 4c,d), autofluorescence is significantly reduced, resulting in a higher contrast Raman spectrum despite lower overall signal intensity. Untreated spheroids (Figure 4c) maintain distinct spectral peaks, while treated spheroids (Figure 4d) exhibit a greater reduction in Raman intensity, reinforcing evidence of biochemical alterations post-treatment. The stronger spectral response at 532 nm confirms its higher Raman scattering efficiency, while 660 nm provides cleaner molecular contrast due to reduced fluorescence interference. These results highlight the trade-off between signal strength and fluorescence suppression in Raman light sheet microscopy.

**Figure 5:**
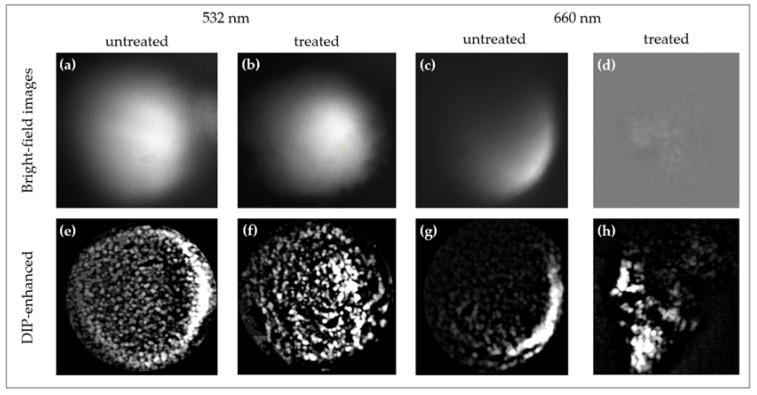
Raman bright-field images for treated and untreated samples using 532 nm and 660 nm lasers. At 532 nm untreated (**a**), treated (**b**), and at 660 nm untreated (**c**), treated (**d**). DIP-enhanced Raman intensity maps at 532 nm untreated (**e**), treated (**f**), and at 660 nm untreated (**g**), treated (**h**). (Note: The displayed image regions are cropped from the original 635 µm × 635 µm field of view for clarity).

The image (Figure 5a–h) illustrates morphological and biochemical differences in spheroids under 532 nm and 660 nm excitation for both untreated and cisplatin-treated conditions. At 532 nm, untreated spheroids maintain a near-spherical structure, while treated spheroids show contraction and peripheral degradation. At 660 nm, untreated spheroids appear more homogeneous, whereas treated spheroids exhibit increased core degradation, highlighting the deeper penetration of longer wavelengths. Raman-based imaging reveals uniform molecular distributions in untreated spheroids, while treated ones show lipid and protein degradation. Deep Image Prior (DIP) denoising significantly improves signal clarity, reducing noise and autofluorescence, particularly in the 532 nm dataset where fluorescence distortions are prominent. DIP-enhanced maps provide high-fidelity molecular gradients, recovering biochemical details lost in fluorescence-heavy Raman datasets. These findings emphasize the role of excitation wavelength and computational enhancement in achieving high-resolution, noise-free molecular imaging.

**Figure 6:**
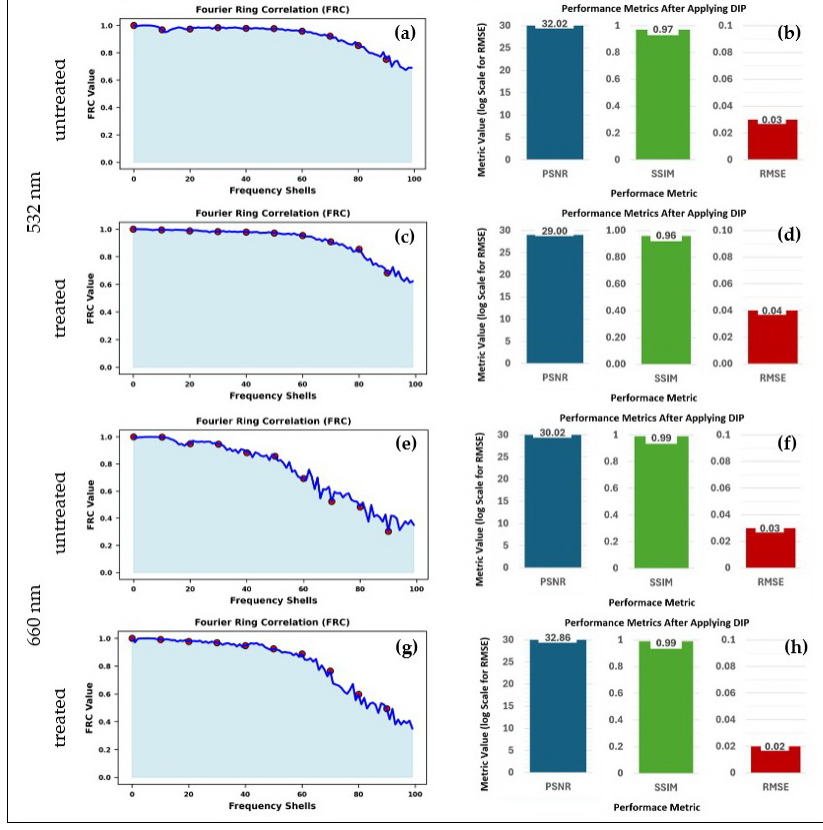
Quantitative evaluation of DIP performance at 532 nm, untreated using FRC metrics (**a**), PSNR, RMSE, SSIM (**b**), and treated FRC metrics (**c**), PSNR, RMSE, SSIM (**d**). Quantitative evaluation of DIP performance at 660 nm, untreated using FRC metrics (**e**), PSNR, RMSE, SSIM (**f**), and treated FRC metrics (**g**), PSNR, RMSE, SSIM (**h**).

To quantitatively assess DIP’s performance, PSNR, RMSE, SSIM, and FRC analyses were conducted (Figure 6a–h). PSNR improvements were observed in both datasets (32.02 dB for 532 nm (untreated 14C) and 30.02 dB for 660 nm (untreated 14C)), confirming enhanced noise suppression. SSIM values increased (0.97 for 532 nm (untreated 14C), 0.99 for 660 nm (untreated 14C)), indicating improved structural fidelity, while FRC-based resolution gains validated DIP’s ability to recover fine molecular features. Similar improvements are observed in the treated 14C samples at 532 nm and 660 nm excitation wavelength, as shown in Figure 6a–h. In Fourier Ring Correlation (FRC) analysis, frequency shells refer to concentric regions in the Fourier domain, each representing a specific range of spatial frequency components. These shells allow quantitative evaluation of image resolution by assessing the similarity between independent image reconstructions at different spatial frequencies. A higher FRC curve across frequency shells indicates better structural preservation and higher image resolution.

The impact of DIP was most pronounced at 532 nm, where autofluorescence suppression was essential for accurate molecular reconstruction. These results establish DIP-enhanced LSRM as a high-fidelity, noise-suppressed framework for Raman imaging in 3D biological models, demonstrating its ability to overcome visible-range autofluorescence limitations while preserving high-resolution biochemical distributions. The comparative evaluation confirms that 660 nm provides deeper penetration with reduced autofluorescence, whereas 532 nm enhances Raman scattering efficiency but requires DIP for fluorescence correction, highlighting the importance of computational enhancement techniques in high-resolution Raman imaging.

### 3.2. Improved 3D Molecular Imaging in Visible-Range Raman Light Sheet Microscopy

**Figure 7:**
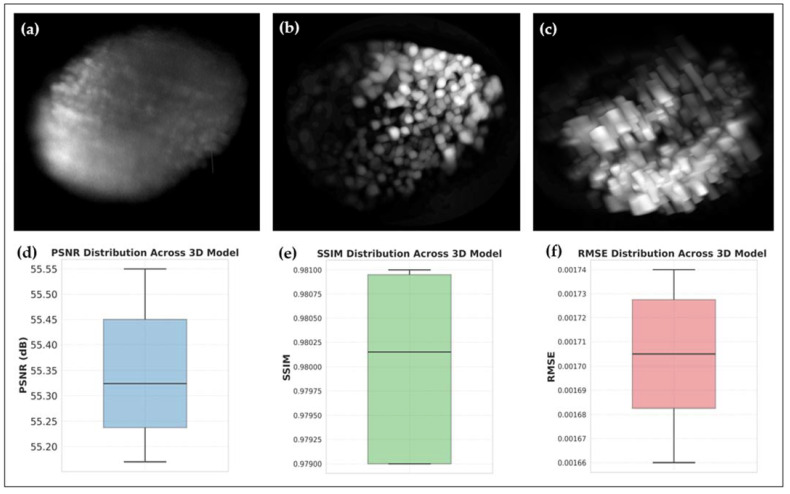
3D Raman reconstruction of HT29 spheroids with fluorescence suppression. (**a**) Original 3D stacked Raman images (41 slices) showing fluorescence artifacts and intensity variations. (**b**) Fluorescence-corrected 3D reconstruction demonstrating improved molecular feature resolution. (**c**) Side-view projection confirming reduced layer-wise fluorescence interference and enhanced structural continuity. (**d**–**f**) Quantitative evaluation of fluorescence suppression using PSNR (**d**), SSIM (**e**), and RMSE (**f**) validating enhanced image fidelity. (Note: The displayed image regions are cropped from the original 635 µm × 635 µm field of view for clarity).

**Figure 8:**
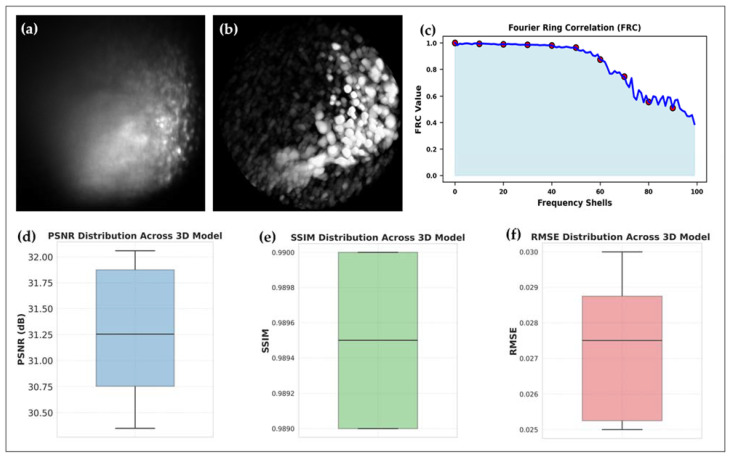
Fluorescence-reduced 3D Raman imaging reconstruction of cisplatin-treated UMSCC-14C spheroids. (**a**) Original 3D stacked Raman images (41 slices) exhibiting fluorescence-related distortions. (**b**) Fluorescence-corrected 3D Raman reconstruction, demonstrating improved molecular feature resolution. (**d**–**f**) Quantitative evaluation of fluorescence suppression using PSNR (**d**), SSIM (**e**), and RMSE (**f**), confirming enhanced volumetric reconstruction. (**c**) FRC analysis, illustrating spatial resolution improvement and reduced fluorescence interference in the 3D dataset. (Note: The displayed image regions are cropped from the original 635 µm × 635 µm field of view for clarity).

To evaluate the impact of visible-range Raman light sheet microscopy in volumetric imaging, 3D Raman models were reconstructed using 41 stacked Raman intensity images of HT29 spheroids (Figure 7) and cisplatin-treated UMSCC-14C spheroids (Figure 8). One of the major challenges in visible-wavelength Raman imaging is the presence of fluorescence interference, which can obscure molecular contrast and affect quantitative biochemical analysis. This study demonstrates how the combination of Raman light sheet microscopy and computational enhancement techniques minimizes fluorescence effects, allowing for artifact-free molecular reconstructions in 3D cellular models.

#### 3.2.1. Volumetric Reconstructions: Pre- and Post-DIP-Enhanced 3D Reconstruction of HT29 and UMSCC-14C Spheroids

Figure 7 presents a comparative analysis of the original and fluorescence-reduced 3D Raman models of HT29 spheroids. The raw 3D reconstruction (Figure 7a) exhibits significant autofluorescence background, leading to intensity variations across layers and reducing contrast in molecular distributions. The fluorescence-corrected model (Figure 7b) demonstrates substantial improvements in molecular uniformity, enhanced contrast, and improved feature delineation. Both panels (a) and (b) are shown from the same viewpoint to enable direct visual comparison of structural improvements. Side-view projections (Figure 7c) confirm that fluorescence-induced artifacts are significantly reduced, ensuring spatial coherence across all layers of the spheroid. All 3D renderings presented in Figure 7 are maximum intensity projections along the Z-axis. These images have also been cropped for better visualization of the central region of the spheroid. Figure 7c is shown from a side view to highlight axial consistency in fluorescence suppression. The stripe-like artifacts visible in this projection are not due to sample deformation but are a result of the interpolation process used during volumetric rendering. Since the model is reconstructed from discrete 2D slices, interpolation along the Z-axis can elongate structures, making spherical regions appear cylindrical. This effect is a common artifact of 3D reconstruction and does not reflect an underlying biological or imaging limitation.

To quantify these improvements, PSNR, SSIM, and RMSE were generated for all 41 slices (Figure 7d–f): PSNR (Figure 7d): Higher values of PSNR indicate improved signal-to-noise ratio, demonstrating the effectiveness of fluorescence suppression. SSIM (Figure 7e): The increased structural similarity confirms that molecular features are well-preserved while reducing unwanted fluorescence artifacts. RMSE (Figure 7f): The decrease in RMSE values further validates the improved accuracy of Raman intensity reconstructions, ensuring artifact-free molecular distributions.

#### 3.2.2. Fluorescence-Reduced 3D Raman Imaging of Cisplatin-Treated UMSCC-14C Spheroids

To further evaluate fluorescence suppression in Raman light sheet-based volumetric imaging, a 3D model was reconstructed for cisplatin-treated UMSCC-14C spheroids (Figure 8). The raw 3D model (Figure 8a) exhibits strong fluorescence interference, leading to signal distortions and inconsistencies in molecular mapping. The corrected 3D model using a combination of processing techniques and the DIP algorithm (Figure 8b) significantly reduces fluorescence background, restoring contrast and improving biochemical precision in the volumetric dataset.

To further quantify fluorescence suppression, PSNR, SSIM, RMSE, and FRC were computed across all 41 slices (Figure 8c–e): FRC (Figure 8c): The FRC analysis confirms a clear enhancement in spatial resolution, validating the improved fidelity of 3D Raman light sheet reconstructions. PSNR (Figure 8d): The increase in PSNR values confirms that fluorescence artifacts have been suppressed, improving overall signal clarity. SSIM (Figure 8d): The structural similarity metric demonstrates that molecular details are preserved while fluorescence background is minimized. RMSE (Figure 8e): A reduction in RMSE indicates improved pixel-wise accuracy, ensuring precise molecular reconstructions.

These findings establish that visible-range Raman light sheet excitation, when combined with fluorescence suppression, provides high-resolution, fluorescence-free 3D molecular imaging, enabling accurate structural and biochemical characterizations. This approach ensures that Raman imaging in the visible spectrum is not limited by fluorescence interference, making it a reliable tool for quantitative volumetric imaging in biological models.

### 3.3. Evaluating the Role of Pre- and Post-Processing in 3D Raman Light Sheet Reconstruction

**Figure 9:**
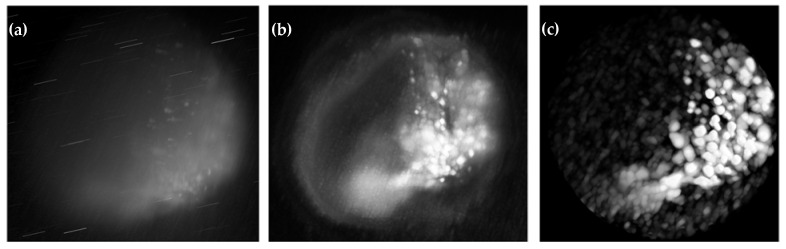
Impact of preprocessing on 3D Raman light sheet reconstruction of UMSCC-14C spheroids. (**a**) Original 3D reconstruction, (**b**) 3D reconstruction after DIP without pre- and post-processing, and (**c**) 3D reconstruction after DIP with pre- and post-processing (Note: The displayed image regions are cropped from the original 635 µm × 635 µm field of view for clarity).

To assess the effect of pre- and post-processing on fluorescence suppression and molecular contrast, a 3D Raman model of cisplatin-treated UMSCC-14C spheroids was reconstructed before and after pre- and post-processing prior to DIP processing (Figure 9). In previous cases, DIP was applied after post-processing, which included median filtering and band-pass filtering, effectively minimizing fluorescence artifacts. Here, DIP was also applied without post-processing, allowing a direct comparison of fluorescence effects in unprocessed Raman light sheet data.

Figure 9a shows the original 3D Raman reconstruction before any computational processing. Figure 9b presents the model after DIP without post-processing, where fluorescence artifacts and spectral distortions remain prominent, reducing molecular contrast and structural clarity. In contrast, Figure 9c shows the model after DIP with post-processing demonstrating reduced background noise, enhanced molecular contrast, and improved Raman-specific feature resolution.

These findings confirm that preprocessing significantly enhances the effectiveness of DIP in visible-range Raman light sheet microscopy, ensuring accurate molecular imaging and artifact-free 3D reconstructions.

### 3.4. Complementary Modalities: Rayleigh and Fluorescence Imaging

**Figure 10:**
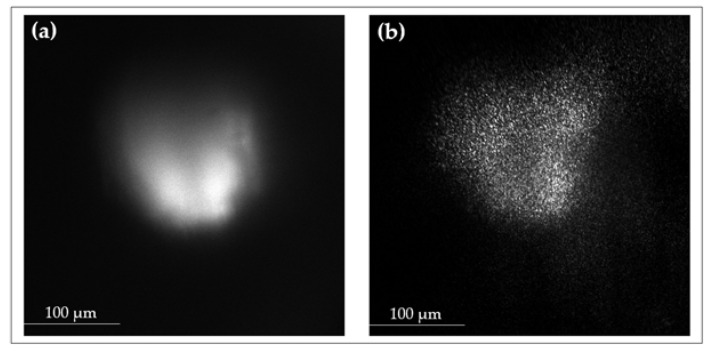
(**a**) Fluorescence image of 14C treated sample using 660 nm excitation laser at 15 mW power and AOTF at 694 nm; (**b**) Corresponding Rayleigh image of same 14C treated sample using 660 nm excitation laser at 1 mW power and AOTF at 650 nm.

This section presents the original images captured directly from the measurement setup, highlighting the system’s capability to acquire Raman, Rayleigh, and fluorescence images. While Raman imaging remains the primary focus, the system also enables Rayleigh and fluorescence imaging, providing additional structural and viability information. Rayleigh images reveal variations in scattering intensity, correlating with Raman-identified necrotic zones, suggesting extracellular matrix degradation. An example of this multi-modal capability is shown in Figure 10, where Figure 10a presents a fluorescence image of a treated UM-SCC-14C spheroid, highlighting metabolic activity and structural integrity, while its corresponding Rayleigh image at 660 nm with 1 mW laser power reveals scattering-based morphological contrast. These unprocessed images provide direct insights into the optical properties of the sample, supporting the interpretation of Raman spectra and biochemical distributions. The integration of Raman, Rayleigh, and fluorescence imaging within a single experimental framework ensures a comprehensive analysis of structural and molecular alterations in 3D spheroids. The ability to capture high-resolution, artifact-free images directly from the setup establishes a robust platform for studying therapeutic effects and advancing cancer research methodologies.

## 4. Discussion

This study presents an optimized light sheet Raman microscopy (LSRM) platform integrating visible-wavelength excitation, preprocessing, and deep learning-based enhancement for high-resolution 3D molecular imaging. By employing 532 nm and 660 nm excitation, the system balances Raman scattering efficiency, fluorescence suppression, and penetration depth, allowing precise molecular characterization in spheroids, including cisplatin-induced biochemical alterations.

### 4.1. Advancing Raman Imaging with Visible-Wavelength Excitation

Visible-wavelength lasers, such as 532 nm and 660 nm, offer significant advantages over traditional near-infrared (NIR) systems by leveraging higher Raman scattering cross-sections, leading to stronger signal intensities and reduced acquisition times. This enhancement is particularly beneficial for imaging heterogeneous 3D cell cultures where high sensitivity is critical. However, visible excitation is not without limitations, such as increased autofluorescence and phototoxicity, which can obscure subtle molecular details and pose challenges for prolonged imaging sessions. These limitations were mitigated by integrating dual-wavelength excitation with precise spectral filtering via acousto-optic tunable filters (AOTFs), ensuring robust spectral acquisition. Nevertheless, achieving high-fidelity, artifact-free imaging required advanced computational solutions to address residual noise and artifacts. Additionally, acquisition times varied significantly depending on the laser source. Furthermore, 785 nm excitation required nearly 6 min to capture a full 41-image stack, whereas 532 nm excitation reduced the acquisition time to approximately 32 s, demonstrating a significant improvement in imaging efficiency. These trade-offs highlight the need for excitation wavelength optimization to balance signal strength, fluorescence suppression, and volumetric imaging depth for high-fidelity Raman-based biochemical mapping.

### 4.2. DIP Algorithm: Overcoming Limitations of Visible Excitation

To mitigate the fluorescence-induced distortions associated with visible Raman excitation, a structured preprocessing pipeline including median filtering and spectral band-pass filtering was employed to improve spectral integrity. Additionally, DIP-based denoising further refined volumetric reconstructions, leveraging self-supervised deep learning to suppress noise while preserving molecular contrast and spatial integrity. This approach significantly reduced background noise, leading to a PSNR improvement of approximately ~50% for both the 660 nm and 532 nm excitation wavelengths compared to raw images. The effectiveness of DIP was confirmed by a 10% increase in SSIM for the 660 nm excitation wavelength and ~32% for the 532 nm excitation wavelength and a corresponding decrease in RMSE values, demonstrating its ability to restore fine structural details while maintaining biochemical specificity. Additionally, Fourier Ring Correlation (FRC) analysis further validated the enhancement in spatial resolution post-processing. A quantitative comparison of PSNR, SSIM, and RMSE values before and after DIP-based denoising is presented in Table 4, illustrating the significant improvements achieved:

### 4.3. Biochemical and Structural Insights from Cisplatin-Treated Spheroids

The platform’s ability to resolve biochemical and structural alterations induced by cisplatin treatment underscores its potential for therapeutic evaluation. Raman imaging revealed decreases in lipid-associated and amide I peaks, alongside increases in phosphate backbone signals, correlating with protein degradation, membrane disruption, and DNA crosslinking—hallmarks of cisplatin cytotoxicity. Volumetric reconstructions enhanced by DIP provided spatially resolved views of necrotic gradients revealing concentric zones of drug-induced cytotoxicity. These findings were corroborated by complementary Rayleigh scattering and fluorescence imaging, offering a multimodal perspective on cellular responses.

### 4.4. Comparative Performance and Multi-Modal Integration

Integrating Raman, Rayleigh, and fluorescence imaging provided complementary insights into molecular and structural variations. Rayleigh scattering imaging confirmed optical density changes, correlating with Raman spectral alterations, while fluorescence imaging validated necrotic regions. Compared to NIR-excited Raman systems, LSRM demonstrated higher signal intensity and reduced acquisition times, despite the fluorescence challenges of visible excitation. The synergy of excitation optimization, preprocessing, and deep learning-based enhancement ensures artifact-free volumetric reconstructions, advancing high-precision Raman imaging in biological systems.

### 4.5. Broader Implications and Future Directions

The proposed system offers broad applications in cancer diagnostics, tissue engineering, and drug discovery, with potential expansion to organoid and patient-derived xenograft models for translational research. While visible-wavelength excitation increases phototoxicity risks, optimized laser parameters mitigate this limitation. DIP, despite its superior denoising capabilities, remains computationally intensive, necessitating GPU-based acceleration for real-time applications.

### 4.6. Challenges and Limitations

Visible-wavelength excitation, while advantageous for enhancing Raman scattering efficiency, increases the risk of phototoxicity during extended imaging sessions, necessitating careful optimization of laser parameters. Moreover, DIP, despite its superior denoising capabilities, is computationally intensive, which may limit its real-time application. Future iterations of the platform will focus on hardware acceleration and algorithmic refinements to address these challenges, ensuring scalability for high-throughput and real-time imaging workflows.

## 5. Conclusions

This study presents a multi-modal light sheet Raman microscopy (LSRM) platform integrating visible-wavelength excitation, structured preprocessing, and deep learning-based enhancement for high-resolution 3D molecular imaging. By leveraging 532 nm and 660 nm excitation, the system balances Raman scattering efficiency, fluorescence suppression, and penetration depth, enabling precise biochemical characterization of cisplatin-induced alterations in spheroids.

A structured preprocessing pipeline incorporating median filtering and spectral band-pass filtering effectively mitigated autofluorescence and background noise, enhancing spectral fidelity. The Deep Image Prior (DIP) algorithm further improved volumetric reconstructions by suppressing noise and diffraction artifacts while preserving structural integrity. The integration of Raman, Rayleigh, and fluorescence imaging modalities provided a multi-scale framework linking molecular-level biochemical perturbations to macroscopic structural changes in treated spheroids.

By overcoming key limitations of conventional Raman imaging, this approach establishes a scalable and high-fidelity platform for volumetric molecular imaging. The combination of excitation wavelength optimization, computational denoising, and multi-modal data fusion enhances sensitivity, spatial resolution, and biochemical specificity. These advancements position LSRM as a powerful tool for biomedical research, particularly in precision oncology, tissue engineering, and high-throughput drug screening.

## Figures and Tables

**Table 1:** Components list for Raman light sheet microscope.

No.	Component Specification	Manufacturer
	Illumination Components	
1	Gem Laser 532 nm, adjustable laser power 0.5–2000 mW	Novanta Inc.
2	LuxX Laser 660 nm, adjustable laser power 0.5–130 mW	Omicron GmbH
3, 4, 8	Broadband mirror, Ø25.4 mm, EO2 coated, mounted in Polaris K1 Kinematic Mirror Mount	Thorlabs GmbH (Lübeck, Germany)
5, 6	Broadband mirror, Ø25.4 mm, EO3 coated, mounted in Polaris K1 Kinematic Mirror Mount	Thorlabs GmbH
7	BrightLine laser dichroic beam splitter, 25.2 × 36.6 mm, reflection band 350–532 nm, transmission band 545.3–1200 nm	Semrock (New York, NY, USA)
9	Mounted achromatic doublet lens, Ø12.7 mm, focal length 25 mm, anti-reflex coating 400–1100 nm	Thorlabs GmbH
10	Mounted achromatic doublet lens, Ø12.7 mm, focal length 50 mm, anti-reflex coating 400–1100 nm	Thorlabs GmbH
11	Mounted cylindrical achromatic doublet lens, Ø25.4 mm, focal length 50 mm, anti-reflex coating 350–700 nm	Thorlabs GmbH
12	UMPLFLN10XW water dipping objective, magnification 10×, numerical aperture 0.3, working distance 3.5 mm	Evident (Hamburg, Germany)
	** Detection Components **	
13	UMPLFLN20XW water dipping objective, magnification 20×, numerical aperture 0.5, working distance 3.5 mm	Evident
14	Acousto-Optic Tunable Filter (AOTF), spectral range 400–1000 nm	Brimrose
15	Polarization Filter	Thorlabs GmbH
16 16a 16b 16c 16d 16e 16f	6-position motorized filter wheel: from position 1 to 6 in 16a–16f Filter 1: Longpass filter, 660 nm Filter 2: Notch filter, 660 nm Filter 3: Bandpass filter, 532 nm Filter 4: Shortpass filter, 660 nm Filter 5: No filter Filter 6: Longpass filter, 532 nm	Thorlabs GmbH Semrock Semrock Semrock Semrock / Semrock
17	Tube lens U-TLU and C-mount (U-TV0.5XC-3)	Evident
18	Aspheric condenser lens, Ø25 mm, focal length 20.1 mm, anti-reflex coating	Thorlabs GmbH
19	sCMOS camera ORCA Flash 4.0 LT+	Hamamatsu (Herrsching, Germany)
20	CXY1 two-axis translating lens mount, Ø550 µm optic fiber	Thorlabs GmbH
21	USB-4D stage (X, Y, Z, R)	Picard-Industries (Albion, NY, USA)
22	Sample chamber, aluminum mounting frame, acrylic water chamber	CeMOS Research and Transfer Center (Mannheim, Germany)
23	MultiSpec^®^Raman spectrometer	tec5 GmbH (Steinbach, Germany)
24	Kaiser spectrometer	Kaiser Optical Systems (Germany)

**Table 2:** Parameters Selection in Measurement Setup for testing.

Incident Wavelength	Modality	Optical Power	Exposure Time	AOTF Wavelength	Filter Wheel Selection	Detected Wavelength
532 nm	Rayleigh Scattering	1 mW	100 ms	522 nm	No filter	532 nm
	Raman Scattering	350 mW	400 ms	631.6 nm	Longpass filter, 532 nm	631.6 nm
660 nm	Rayleigh Scattering	1 mW	100 ms	650 nm	No filter	660 nm
	Raman Scattering	130 mW	5000 ms	815 nm	Longpass filter, 660 nm	815 nm

**Table 3:** Raman peak positions and vibrational modes.

Peak Position/cm^−1^	Vibrational Mode	References
3000–3400	CH stretch	[27]
2880–2895	CH_2_ asymmetric stretching	[28]
2929–2937	CH_3_ stretching	[29]

**Table 4:** Quantitative Evaluation of Image Enhancement Using DIP by using an 14C Treated Spheroid Image.

Excitation Wavelength	Pre- and Postprocessing	PSNR (dB)	SSIM	RMSE
532 nm (Raw)	No	19.56	0.723	0.105
532 nm (After DIP)	Yes	29.00	0.958	0.0354
660 nm (Raw)	No	21.08	0.898	0.088
660 nm (After DIP)	Yes	31.86	0.989	0.026

## Data Availability

The data used to support the results of this study are included within the article. In addition, some of the data in this research are supported by the references mentioned in the manuscript. If you have any queries regarding the data, the data of this research is available from the corresponding author upon request.
